# Border-ownership reversals determine the strength of metacontrast masking

**DOI:** 10.3758/s13414-026-03339-z

**Published:** 2026-09-24

**Authors:** Doris E. Dijksterhuis, Nina Vreugdenhil, Pieter R. Roelfsema, Matthew W. Self

**Affiliations:** 1 Department of Vision and Cognition, Netherlands Institute for Neuroscience (KNAW), Amsterdam, the Netherlands; 2 Department of Integrative Neurophysiology, VU University, Amsterdam, the Netherlands; 3 Department of Psychiatry, Academic Medical Centre, Amsterdam, the Netherlands; 4 Laboratory of Visual Brain Therapy, Sorbonne Université, Institut National de La Santé Et de La Recherche Médicale, Centre National de La Recherche Scientifique, Institut de La Vision, 75012 Paris, France; 5 School of Psychology and Neuroscience, University of Glasgow, 62 Hillhead St., Glasgow, G12 8QQ Scotland

**Keywords:** Visual perception, Border-ownership, Metacontrast masking, Figure–Ground perception, Gestalt

## Abstract

Visual objects can be rendered invisible if their borders are assigned to other objects, showing that border-ownership (BO) is critical for perception. Objects can also be rendered invisible by using masking to limit the amount of time the brain has to process the object. An example of this is metacontrast masking (MM). MM occurs when an object is briefly presented and is followed by the presentation of a mask with closely adjacent contours. The mask can render the target object invisible, even though the target object is processed in high level brain areas. We propose that a critical factor that determines whether the target is perceived or not, is the amount of time that the target object retains ownership of its borders. What many MM paradigms have in common, is that when the mask appears, it reverses the directions of ownership at the borders of the previously presented target object. We hypothesized that this reversal in BO disrupts recurrent processing of the target object and impair perception. Here we tested this hypothesis by designing new MM paradigms with masks that either reverse ownership of the borders of the target object or not. We consistently found that masking was stronger when the mask gained ownership of the borders of the target object than when border ownership was unaffected by the mask. Our results indicate that prolonged ownership of borders is crucial for creating a stable model of the world, which enables the perception of stimuli.

## Introduction

**Fig. 1:**
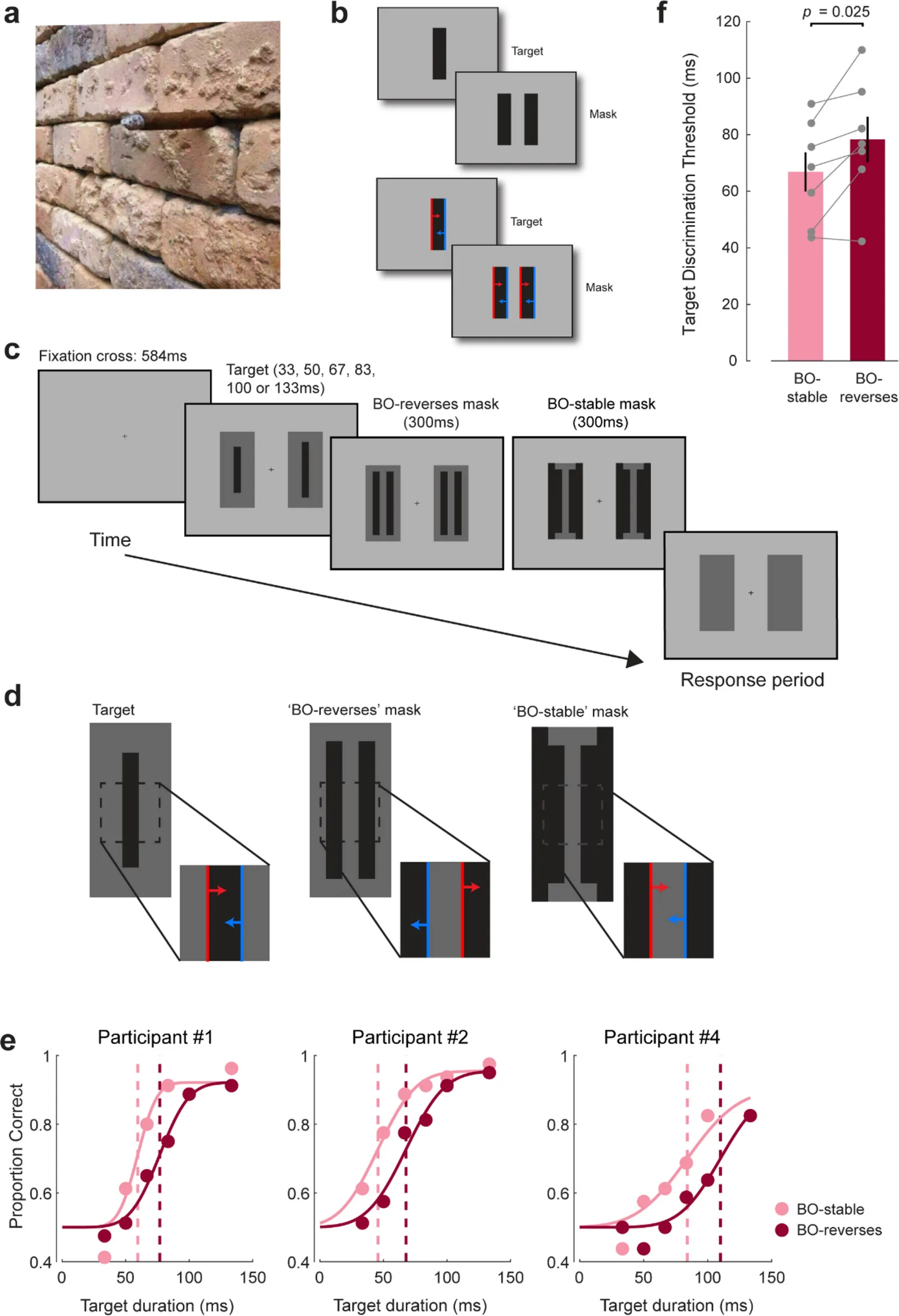
Experiment 1a: BO reversals lead to stronger masking. **a** Illusion demonstrating the importance of BO in establishing a visual percept. There is an object hidden in the image (see Fig. 5a for the answer). **b** (Upper row) Typical MM paradigm. (Lower row) Target and mask stimuli are shown with arrows and colours indicating BO direction. When the mask appears, the BO direction switches at the target–mask boundary. **c** Schematic depiction of a trial sequence. The participant’s task was to press a key to signal the location of the longest target bar. **d** Border-ownership during target and mask presentation. The arrows and colours indicate the direction of the BO. **e** Example data from three representative participants. The solid lines show psychometric functions fitted to the data. The dashed lines indicate the target discrimination threshold. **f** Results of Experiment 1a. The bars represent the mean discrimination thresholds per masking condition across seven participants. The error bars indicate ± 1 *SEM*. The dots represent the discrimination thresholds per participant. The *p* value is from a two-sided paired *t* test. (Colour figure online)

We live in a complex 3D environment where nearby objects partially occlude more distant objects. Borders between visual objects need to be correctly assigned to the foreground object to make sense of 3D structure and to correctly process an object’s shape (Nakayama, 1996; Nakayama et al., 1989; Rubin, 1921). Border-ownership (BO) assignment has profound effects on the contents of our perception. This was first elegantly demonstrated by the Gestalt psychologists—for example, in Rubin’s Face–Vase illusion, in which perception becomes repeatedly reorganised into faces or a vase depending on the assignment of the border between the image regions (Rubin, 1915). The process of border assignment is intimately related to the process of figure–ground segregation as regions that lose possession of their borders typically become assigned to the background (Rubin, 1921). These background regions are more difficult to recognise and are less strongly encoded into memory (Driver & Baylis, 1996). In extreme cases, assignment of a visual region to the background can result in a complete failure to detect an otherwise salient object in the visual scene. This is demonstrated by the illusion in Fig. 1a. There is an object hidden in the image (the answer is in Fig. 5a). For most people the visual system incorrectly assigns the borders of the object to the surrounding bricks and the object only becomes visible after instruction about the presence of the object. The illusion suggests that BO assignment is necessary for the detection of an object in a visual scene. At the neural level, mid-tier visual areas such V2, V3, and V4 contain large populations of cells whose activity strongly correlates with the perceptual assignment of borders (Franken & Reynolds, 2021; Hesse & Tsao, 2016; Jeurissen et al., 2024; Qiu & von der Heydt, 2005, 2007; Zhou et al., 2000). Correct activation of these BO tuned cells, which not only encode borders between image regions, but also which image region owns the border, may then be critical for correct figure–ground assignment and perception of 3D structure in a visual scene. In this study we propose that BO assignment is also one of the key principles at play in another well-known perceptual phenomenon, that of meta-contrast masking (MM).

MM occurs when the visibility of a briefly presented target is reduced or abolished by a subsequently presented mask whose borders are closely adjacent to the target (Breitmeyer & ÖĞmen, 2006). A typical paradigm would consist of a briefly presented target bar which is followed at varying delays (stimulus onset asynchrony [SOA]) by a mask consisting of two flanking bars (see example in Fig. 1b). If we consider the ownership of borders during this paradigm (blue and red arrows in Fig. 1b) the borders of the black target bar are initially assigned to the region occupied by the target. When the mask appears, the direction-of-ownership of these borders reverses and they become assigned to the two bars in the mask. In other words, the mask gains ownership of the target’s borders and the region that was previously occupied by the target now becomes assigned to the background in perception. The gain-of-ownership of the target borders by the mask is common to many MM paradigms and could in principle be a key mechanism which reduces the perceptual strength of the target. Previous studies have suggested that backwards masking may result from interference between the strong, transient activity generated in V1 by the onset of the mask and the arrival of reentrant feedback processes related to the target in V1. If the mask-onset activity disrupts the recurrent processing of the target this could lead to weaker perception (Fahrenfort et al., 2007; Lamme et al., 2002). This would be consistent with the finding that masking is typically strongest when the onset of the mask is delayed relative to the onset of the target by ~ 30–100 ms (Kahneman, 1967) as it takes approximately 50 ms before recurrent signals arrive back in V1 from higher visual areas. We propose that sustained, recurrent interactions between BO-tuned cells and cells in V1 are necessary for establishing robust perception of the target stimulus (Jeurissen et al., 2024). We therefore predict that masks which switch ownership of the target’s borders will disrupt this recurrent process and lead to strong reduction in perceptual strength at SOAs of in the 30–100-ms range.

In this study we explored whether the strength of masking in MM paradigms is dependent on whether the mask gains ownership of the targets’ borders in a series of behavioural experiments. We designed two new MM paradigms with two different meta-contrast masks that either gain ownership of the borders of the target object or not. In our MM experiments we consistently show that, when the direction of BO is maintained across the target–mask transition, the target is perceived more strongly, compared to when the mask gains ownership of the borders.

## Experiment 1a

In Experiment 1a we tested whether the strength of MM depends on whether the mask gains ownership of the target’s borders (Fig. 1c). The participants had to indicate which of two target bars was longer (left or right). After the brief presentation of the two bars, a mask appeared that either caused a change in the ownership of the borders of the target (*BO-reverses* condition) or did not (*BO-stable* condition). In the BO-reverses condition, the mask consisted of two flanking black bars, a commonly used meta-contrast mask (Kahneman, 1967; Macknik & Livingstone, 1998). In the BO-stable condition these same black bars were presented, but additional black regions were added to the mask so that the central grey region formed the shape of a letter ‘I’ (Fig. 1d). The ‘I’ region was perceived to be an object by the participants, due to its central position, convex corners and the ability of familiar objects to bias figure–ground assignment (Peterson & Gibson, 1994; Peterson et al., 1991). This ensured that the direction of ownership of the target’s borders remained the same across the target–mask transition. We hypothesized that target discrimination accuracy would be lower in the BO-reverses condition.

### Method

#### Participants

Eight participants took part in the experiment, all were naïve to the aims of the experiment (aged 21–39 years; four were women; all were right-handed). They were employees from the Netherlands Institute for Neuroscience. One participant was excluded as their maximum performance did not reach 75% correct. We obtained ethical approval from the ethics committee at the University of Amsterdam and all methods in the current study were performed in accordance with relevant guidelines and regulations. All participants signed informed consent before participating in the experiment.

#### Stimuli

Stimuli were presented on a Trinitron T5400 monitor with a maximum luminance of 77.52 cd.m^−2^ at a refresh rate of 60 Hz in a dark room. The size of the monitor was 47.6° × 39.2° with a chin rest at a viewing distance of 35 cm. During the entire experiment, a 0.6° × 0.6° black fixation cross was presented on a 33.75 cd.m^−2^ grey background in the middle of the screen. The targets were two black bars sized 0.8° (width) × 7.4° (height) for the shorter bar and 8.6° for the longer bar. They were presented 6.0° left and right from the fixation cross. The longer and shorter bars randomly varied between the left and right side. The BO-reverses mask consisted of two bars of 0.8° × 9.7° with a space of 0.8° between them, and the BO-stable mask was shaped as is shown in Fig. 1c–d. The size of the longer black part of the BO-stable mask was 0.8° × 11.6° and the size of the shorter black part 0.8° × 9.7° with a space of 0.8°. Targets and masks were always black, presented in the centre of a dark grey square of 3.9° × 11.6° with a luminance of 8.55 cd.m^−2^. Stimuli were programmed using the COGENT graphics toolbox developed by John Romaya at the LON at the Wellcome Department of Imaging Neuroscience in MATLAB R2006 (The MathWorks, Natick, MA, USA).

#### Procedure

Each participant first performed a training session in which they learned the task. First, the targets were revealed so that the participants knew what to look for. The task was a two-alternative forced-choice task where the participants had to report whether the longer target was on the left or the right side of the fixation cross. The duration of the target was pseudo-randomly chosen per trial between the following values: 33, 50, 67, 83, 100, and 133 ms. After an interstimulus interval (ISI) of 0 ms, the target was always followed by either the BO-reverses or BO-stable mask, in a pseudo-random sequence. The duration of the mask was always 300 ms, thereafter only the dark-grey square remained. In the training session, the participants received feedback after their response (a green or red dot meaning correct or incorrect, respectively). After the participants confirmed that they were familiar with the task, they performed the real experiment. The experiment started with an introduction and again an example of the target. There was now no feedback and a new trial started after the participant gave a response. The participants responded by pressing the left or right arrow key, indicating the location of the longer target. After every 24 trials, a black screen appeared for 4 s, allowing the participants to briefly rest and reduce adaptation effects. Hereafter, the background grey screen appeared again, which indicated that the participants had 2 s before the next trial started. Each participant completed 960 trials in approximately 30 min.

#### Statistical analysis

We fit cumulative normal functions to the probability of a correct response (ψ) at each target duration x of the form:

$$\psi \left(x,\alpha ,\beta ,\gamma ,\lambda \right)=\gamma +\frac{(1-\gamma -\lambda )\beta }{\sqrt{2\pi }}{\int}_{-\infty }^{x}exp\left(-\frac{{\beta }^{2}{(x-\alpha )}^{2}}{2}\right).$$

A separate function was fit per condition (BO-reverses/stable) using maximum likelihood using the Palamedes toolbox for MATLAB (Prins & Kingdom, 2018). The free parameters were the threshold α, slope β, and lapse-rate λ of the function. The guess rate, γ, was set to 0.5. The lapse rate (i.e., the number of incorrect responses at infinite target durations) was constrained to be the same for both conditions and was constrained to lie between 0 and 0.1. The threshold (i.e., the target duration at which performance was halfway between chance and ceiling performance) was taken as the target discrimination threshold. The significance of the difference in threshold between the two mask types was tested with a two-sided paired-samples *t* test. All statistical analyses were performed in MATLAB.

### Results

As expected, the accuracy of the seven included participants in determining which bar was longer improved with the duration of the target bars. We fit psychometric functions to the accuracy data and estimated target discrimination thresholds as the target duration at which the fitted function was halfway between chance and ceiling performance (Fig. 1e). The accuracy of the fits was generally high across participants, mean *r*^2^ = 0.95 ± 0.05 (*SD*), range: 0.8–0.99. We did not observe any effect of mask type on the slopes of the psychometric functions, *t*(6) = 0.2, *p* = 0.85, paired* t* test (two-sided), suggesting that the difference in BO-reversal did not affect decision uncertainty or sensory noise. However, there was a significant difference between the target discrimination threshold in the BO-reverses condition compared to the BO-stable condition, Fig. 1f; *t*(6) =  − 2.96, *p* = 0.025, paired *t* test (two-sided). In the BO-reverses condition, the target discrimination threshold was 78 ± 8 ms (mean ± *SEM*), which is significantly longer than the threshold of 67 ± 7 ms in the BO-stable condition. This shows that participants required more time to discriminate which bar was longer when the mask reversed the direction of ownership of the target’s borders compared to when it did not. Hence, masks that maintain the direction of ownership are less effective in reducing visibility.

### Discussion

Experiment 1a shows an enhancement of performance when the targets were followed by a mask that maintained the direction of BO at the borders of the targets compared with a traditional metacontrast mask that reversed the direction of BO. The result suggests that some of the masking effect observed using metacontrast masks may be due to this reversal in BO at the target’s borders. We note however an important caveat here. Metacontrast has typically been assessed by varying the SOA between the target and mask and the effects of metacontrast are strongest at intermediate SOAs (30–100 ms) in paradigms that produce Type B masking (Breitmeyer & ÖĞmen, 2006). In this experiment, the target duration was varied, and the mask always followed the target with an inter-stimulus interval of 0 ms. This has the advantage that the target and mask never appeared on the screen at the same time, but it meant that the SOA was determined entirely by the target duration and shorter SOAs therefore had shorter target durations. This relationship did not allow us to investigate the timing of the masking effect and confirm that the masks we used produced Type B masking. To address, this issue, we ran a second experiment, Experiment 1b, in which target duration was held constant and SOA was varied independently.

## Experiment 1b

**Fig. 2:**
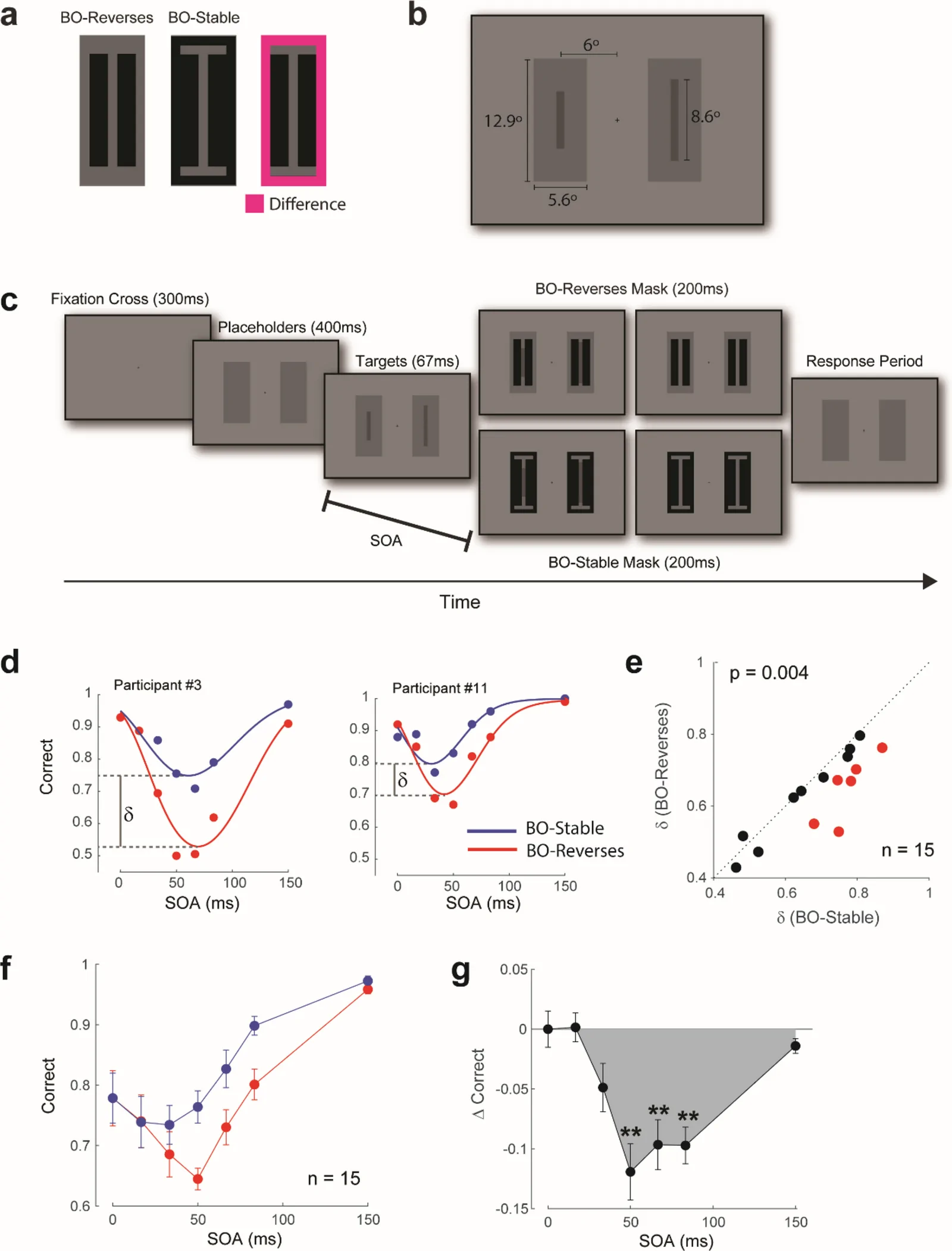
Experiment 1b: Investigation of mask strength with SOA. **a** The two mask types used in Experiment 1b and the pixelwise difference between them (purple). **b** The spatial layout of the targets. **c** Schematic depiction of a trial sequence. The participant’s task was to indicate the location of the longest target bar. **d** Example data from two participants. The solid lines show fits of a polynomial logistic regression. The δ statistic is the difference between the minima of the fitted functions. **e** The δ statistic in the two mask conditions across 15 participants. The red markers indicate participants where the δ statistic was significantly lower in the BO-reverses condition (*p* < 0.05). The *p* value indicated in the figure comes from a paired *t* test. **f** The average performance at each SOA across 15 participants. The error-bars indicate ± 1 *SEM*. **g** The difference in performance between the BO-stable and BO-reverses condition. The asterisks indicate the significance of a two-sided* t* test carried out at each SOA (**p < 0.01, Bonferroni correction for multiple comparisons applied). The error bars indicate ± 1 *SEM*. (Colour figure online)

In Experiment 1b we used similar masks to those described above but now held the target duration constant while varying the SOA in typical ranges for metacontrast paradigms (0–150 ms). We also made several improvements to the paradigm. Firstly, we enlarged the black region around the ‘I’ shaped mask to make perception of the ‘I’ shape more convincing (Fig. 2a) and we confirmed with each participant before the experiment that they perceived the mask as a letter ‘I’. Secondly, we ran a larger cohort of participants (*n* = 16) to improve the power of the design. Lastly, we used a more efficient design in which the target discrimination ability was directly assessed at each SOA without the need for fitting psychometric functions. In other aspects the experimental design was similar to Experiment 1a.

### Method

#### Participants

Sixteen participants took part in the experiment (aged 20–27 years; eight women, eight men; 15 right-handed, one left/mixed-handedness), all were naïve to the aims of the experiment. We obtained ethical approval from the ethics committee of the College of Medical, Veterinary & Life Sciences at the University of Glasgow (#200,240,308), and all methods in the current study were performed in accordance with relevant guidelines and regulations. All participants signed informed consent before participating in the experiment.

#### Stimuli

All stimuli were programmed using Psychtoolbox-3 (Version 3.0.19.16) in MATLAB R2022a (The MathWorks). Stimuli were presented on an LCD monitor (Hewlett-Packard), with a maximum luminance of 264.2 cd.m^−2^ at a refresh rate of 60 Hz in a dark room. The size of the monitor was 59.75 cm × 34 cm with a chin rest at a viewing distance of 49 cm. The monitor had a resolution of 2,560 × 1,440 pixels, subtending 62.7° of visual angle in the horizontal direction and a resolution of 40.8 pixels per degree of visual angle. Each trial began with the presentation of the fixation cross for 300 ms. The cross dimensions were 0.25° × 0.25° and it was presented on a 54.18 cd.m^−2^ grey background. The cross position was jittered between trials in a range of ± 1.5 d.v.a. This was followed by the presentation of two dark-grey (32.6 cd.m^−2^) placeholder rectangles, 5.6° (width) × 12.9° (height), positioned equally spaced to the left and right of the fixation cross at an eccentricity of 6°. After 400 ms, two dark-grey targets appeared (Fig. 2b). The targets had a luminance of 16.9 cd.m^−2^. The longer target always had a width of 0.8° and a length of 8.6°. The shorter bar had the same width, but its length was controlled by a preexperimental staircasing procedure (see below). The bars were presented at 6.0° eccentricity, left and right from the fixation cross. The position of the longer and shorter bars was pseudo-randomly varied between the left and right side. The targets were followed after an SOA of 0, 17, 33, 50, 67, 83 or 150 ms, by either the BO-reverses or BO-stable mask. The BO-reverses mask consisted of two 0.8° × 9.7° black bars, with a distance of 0.8° between the inner borders of the bars, which were presented on either side of fixation so that the inner borders of the bars were precisely aligned with the contours of the target. The BO-stable mask was shaped as is shown in Fig. 2a and c. The size of the longer black part of the BO-stable mask was 0.8° × 11.6° and the size of the shorter black part 0.8° × 9.7° with a space of 0.8°. The duration of the target was always 67 ms and the mask duration was always 200 ms.

#### Procedure

Each participant first performed a training session in which they learned the task. First, the targets were shown so that the participants knew what to look for. The task was a two-alternative forced-choice task where the participants had to report if the longer target was on the left or the right side of the fixation cross (Fig. 2a,c). The participants first practiced trials with a long SOA (333 ms) under the guidance of the experimenter. After the participants confirmed that they were familiar with the task, they performed a brief practice session in which the SOA was decreased. They performed eight trials at SOAs of 267, 167, 100, and 0 ms. If performance was adequate, they moved to a staircasing procedure to set the difficulty of the task. The length of the short bar was used to control difficulty. Using a 3-down, 1-up staircasing procedure, participants performed trials at an SOA of 83 ms with only the BO-reverses mask. Starting at a bar length of 5°, the length was increased after three correct trials and decreased after one incorrect trial. The staircasing was run until 8 reversals in direction were obtained and the final experimental length was taken as the average of the last two reversals. In case the threshold length was less than 6° the staircasing was repeated. After staircasing, the main experiment began. The experiment started with two easy trials of 200-ms SOA, which were excluded from analysis. The participants responded by pressing the left or right arrow key, indicating the location of the longer target. No feedback was given, and a new trial started after the participant gave a response. After every 28 trials, a black screen appeared for 4 s, which was meant for the participants to briefly rest and reduce adaptation effects. Hereafter, the background grey screen appeared again, which indicated that the participants had 2 s before the next trial started. Each participant completed 702 trials in a single session and completed two sessions in total with a short break in between. The entire experimental session lasted approximately 1 h.

#### Data analysis

Data cleaning was performed by removing trials with reaction times of greater than 3 s (max 1.9% of trials). We examined the mean accuracies of each participant, in one participant the staircasing procedure did not work as desired as the average performance across all conditions was close to ceiling at 92.4%. We removed this participant leaving 15 included participants. We initially noticed that several participants had biases to report the bar on the left side of the screen as being longer, particularly at SOAs that produced low performance. We suspect the bias was due to a preference to use the index finger over the middle finger, which manifested during guessing when unsure of the correct answer. Further analysis showed no interaction of this bias with the mask type (BO-stable or BO-reverses) and so all other analyses averaged across trials regardless of the location of the longer bar. As expected, the accuracy of individual participants showed a ‘U-shaped’ relationship with SOA, with performance being more accurate at the shortest and longest SOAs and less accurate at intermediate SOAs (in the 30–100-ms range). Accuracy (correct/incorrect) was modelled using binomial logistic regression. The log-odds of a correct response, logit(p_i_), was modelled as a cubic polynomial function of SOA, with mask type as a categorical predictor interacting with SOA up to quadratic order:

$$logit\left({p}_{i}\right)= \mathrm{log}\left(\frac{{p}_{i}}{1-{p}_{i}}\right)$$

$$logit\left({p}_{i}\right)={\beta}_{0}+ {\sum}_{j=1}^{3}{\beta}_{j}SO{A}_{i}^{j}+ \gamma MaskType + {\sum}_{j=1}^{2}{\delta}_{j}SO{A}_{i}^{j}MaskType,$$

Where MaskType was a dummy-coded variable with values 0 (BO-stable) or 1 (BO-reverses), SOA was treated as a continuous variable, and β, γ, and δ were regression coefficients.

To assess whether there was a significant effect of mask type, we used a relabelling approach in which the condition labels were scrambled at each SOA and surrogate data points were generated. The surrogate data were then fit using the polynomial regression approach described above. For each iteration of the shuffling procedure, we took the difference in the minimum of the fitted functions for each value of MaskType as the statistic (δ). The significance of the veridical value of δ was calculated as the fraction of absolute shuffled values that were greater than, or equal to, the veridically observed values.

For group level analysis we used multiple approaches. Firstly, we used the δ values calculated as described above as descriptive statistics of each participant and compared these using paired-sample *t* tests. We also directly assessed whether the difference between masking conditions at each SOA was different than zero using Bonferroni-corrected two-tailed *t* tests. Finally, we utilized the individual trial responses in a generalized linear mixed-effects model (GLMM) using a logit link function. The data were fit using the following equation:

$$logit\left({p}_{i}\right)\sim 1+{SOA}^{2}*MaskType+{SOA}^{2}+SOA*MaskType+\left(1\left|ParticipantID\right.\right),$$

where the * operator indicates an interaction term and the | operator indicates a random effect. The cubic expression of SOA was chosen as it produced the lowest Bayesian information criteria value. It should be noted that the choice of polynomial order did not affect the conclusions, as similar results were obtained with quadratic and tertiary terms for SOA. The GLMM was fit using the ‘glme.m’ function in MATLAB using the Laplace approximation to maximum likelihood. The significance of the MaskType predictor was assessed using a likelihood ratio test comparing the full model with a nested model excluding all terms containing the MaskType predictor.

### Results

The accuracy of individual participants in identifying which of the target bars was longer showed a U-shaped relationship with SOA as is typically observed in Type B MM paradigms (Fig. 2d). Two example participants are shown in Fig. 2d. Accuracy with the BO-reverses mask was high at the shortest and longest SOAs but reached its minimum at intermediate SOAs. The example participants showed strong masking in the 50–100-ms SOA range, with performance reaching chance levels for the BO-reverses condition in Participant 3. However, masking was much weaker for the BO-stable condition. We fit the data using polynomial regression (see Methods) and observed strong interactions between SOA and mask type in both participants. As the exact shape of the masking function varied between participants and the regression analysis revealed significant interactions with both the quadratic and linear SOA terms we derived a simpler measure of masking strength, as the minimum of the fitted regression curve in the SOA range 0–150 ms. We observed that, in these example participants, the difference in the minimum of the fitted function (δ) was significantly greater than would be expected by chance as assessed by a label-shuffling approach (see Methods). Across the 15 included participants we generally saw lower performance minima in the BO-reverses condition, and this difference was significant in six out of 15 participants (Fig. 2e). Averaged across all participants, accuracy patterns showed a clear dip at intermediate SOAs (Fig. 2f). There was no difference between the two masking conditions for the first two SOAs (0, 17 ms), but a difference began to arise at around 33 ms, becoming significant (*p* < 0.01, Bonferroni-corrected *t* tests) for the 50, 67, and 83-ms SOAs (Fig. 2g). Generalized linear mixed effects analysis, which control for accuracy offsets in different participants, revealed significant interactions between SOA^2^ and mask type as well as SOA and mask type. Including mask type in the model produced significantly better fits (Likelihood Ratio Test, ∆LogLikelihood = 159, ∆d.o.f = 3, *p* < 10^–10^). The simpler analysis of the difference in the fitted minima of individual participants (δ) also showed a significant difference between the two masking conditions (paired *t* test, *p* = 0.004). The results indicate that there was a strong, classical metacontrast masking effect for the BO-reverses condition that was considerably weakened when the mask was shaped so that BO relationships remained stable across the target–mask transition.

### Discussion

Experiments 1a and 1b suggest that stable direction of border assignment at the target–mask boundary is beneficial for visibility. In both experiments the BO-reverses mask, which is a traditional metacontrast mask, resulted in lower target discriminability, whereas the BO-stable mask, that preserved BO direction, had less effect. The BO-reverses mask showed a typical Type B masking profile with the strongest masking observed in the 30–100-ms range. The BO-stable mask reduced the strength of masking at these same SOAs while having no effect at the shortest and longest SOAs. The results indicate that the change in BO-arrangement reduced the strength of Type B metacontrast masking.

In Experiment 1b, we varied SOA while keeping target duration constant. This results in SOAs where the target and mask overlap temporally. At these SOAs, and particularly at the 0-ms SOA where the target and mask appear simultaneously, the BO assignment of the target is ambiguous. The target–mask combination may be perceived as two separate objects, in which case the target and mask compete for assignment where the two regions meet, or they may be combined into a grouped percept where the target appears to be part of the mask. At longer SOAs, the prior appearance of the target should bias perception so that the target is perceived as an independent object. This more complex border–assignment relationship is unavoidable in paradigms that vary SOA, but we note that similar results were obtained in Experiment 1a where the target and mask never appeared on the screen simultaneously, suggesting that this complexity is not critical for the results.

We note two further caveats here. Firstly, the shape of the mask differed across the two conditions: the mask in the BO-stable condition had a larger black region than in the BO-reverses condition, and consequently a lower mean luminance. It is conceivable that the increased masking strength in the BO-reverses condition may have been related to this difference in luminance flux. Although we note here that theories of masking which relate masking strength to the change in energy for the mask (Alpern, 1953; Weisstein, 1972) would predict the opposite result—that is, stronger masking in the BO-stable condition. Nevertheless, we designed Experiment 2 to try to minimise the physical differences between the two mask types as much as possible. Secondly, our hypothesis is that stable BO assignment is crucial for perception of objects, but in Experiment 1 we used a length-judgement test, which may have been sensitive to other features than the visibility of the bars. In Experiment 2 we switched to using a judgement of the contrast of the target, which is more directly related to target visibility.

## Experiment 2

**Fig. 3:**
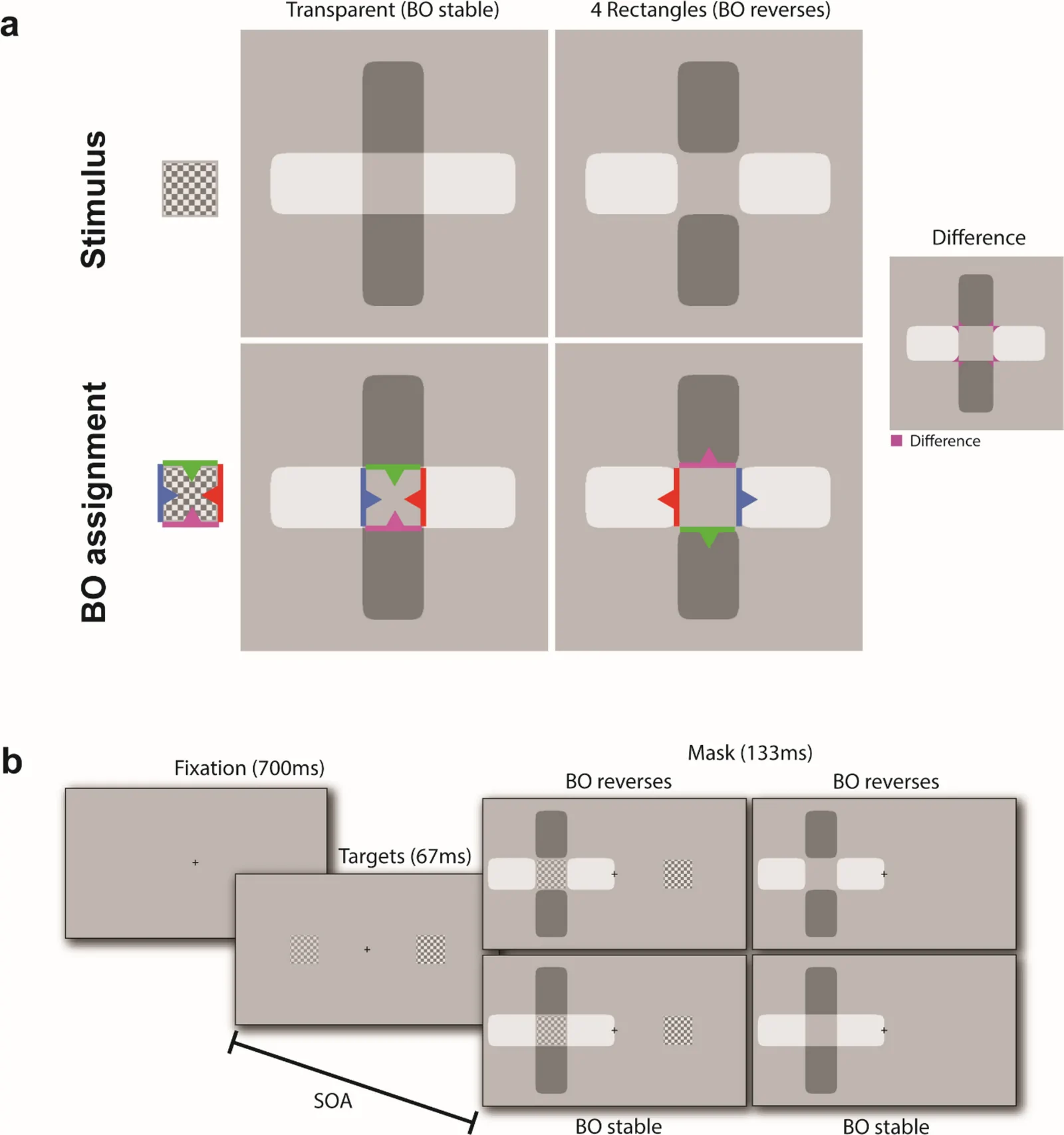
Controlling BO reversals with near identical masks. **a** (Upper row) The transparent bar illusion was used to create two mask types in Experiment 2. People generally perceive the mask on the left as two transparent overlaid bars. A small adjustment to this stimulus, rounding the inner corners, causes the percept to shift to four separate rectangles. The pixelwise difference between the masks is shown by the purple pixels on the far right. (Lower row) The BO assignment of the central square in the two masks, note how the BO direction is reversed between the masks. **b** Schematic depiction of a trial sequence. The participant’s task was to press a key to signal the location of the checkerboard target with the highest contrast. (Colour figure online)

Experiment 1a–b demonstrated that masks that do not reverse the BO assignment of the target region are less effective than traditional masks that do reverse these relationships. In Experiment 2, our primary aim was to design masks that do, or do not, switch the BO assignment of the target while keeping the masks as physically similar as possible. To achieve this goal, we took advantage of a paradigm first developed by the von der Heydt group using overlapping transparent bars (Fig. 3a). Qiu and von der Heydt (2007) showed that the activity of BO cells in V2 of macaque monkeys reflects the perceived organisation of these bars. Critically, the perception of the transparent bars can be switched into four separate rectangles by making a very minimal change to the inner corners of the rectangles (Fig. 3a). This results in two different stimulus configurations which have opposite BO assignments with very minimal physical differences between them. We used the transparent (BO-stable) and four-rectangle (BO-reverses) stimuli as masks in this experiment.

We also changed the task to a contrast judgement task to more directly relate the masking effects to stimulus visibility. The targets in this experiment were two checkerboard patterns. One checkerboard (the ‘test’ stimulus) had a constant contrast and was presented at the centre of the masked region whereas the other checkerboard (the ‘reference’ stimulus) could vary in contrast and was presented in isolation in the opposite hemifield (Fig. 3b). Participants were asked to judge which checkerboard was higher in contrast and we used the method of constant stimuli to estimate the point of subjective equality (PSE) and therefore the perceived contrast of the test checkerboard. We theorized that the BO-reverses four-rectangle mask should reduce the perceived contrast of the target at intermediate SOAs whereas the BO-stable transparent mask should have a weaker effect.

### Method

#### Participants

Sixteen participants took part in the experiment (aged 21–29 years; eight women, eight men; 13 right-handed, three left/mixed-handedness), all were naïve to the aims of the experiment. We obtained ethical approval from the ethics committee of the College of Medical, Veterinary & Life Sciences at the University of Glasgow (#200,240,308) and all methods in the current study were performed in accordance with relevant guidelines and regulations. All participants signed informed consent before participating in the experiment.

#### Stimuli

Stimuli were generated using the same system as in Experiment 1. Each trial began with the presentation of the fixation cross for 700 ms. The cross dimensions were 0.25° × 0.25° and it was presented on a 132.2 cd.m^−2^ grey background. The position of the cross was jittered between trials in a range of ± 1.5 d.v.a. The targets were checkerboards and each check was 0.2° × 0.2°. The checkerboard had the same mean luminance as the background grey. The checkerboard had a small gap around its edges of 0.1° to help maintain the transparency illusion for the transparent mask. The constant-contrast checkerboard had a Michelson contrast of 37.8%, and the variable-contrast checkerboard was shown at one of seven contrasts, equally spaced from 15.1% to 60.5%. The targets were followed after an SOA of 0, 17, 33, 50, 67, 83, or 133 ms, by either the four-rectangle BO-reverses or transparent BO-stable mask. Both masks contained four rectangular regions; two lighter grey (212 cd.m^−2^) and two darker grey (52.2 cd.m^−2^). The position of the light and dark grey regions was counter-balanced across trials. The sizes of the regions were 2° (width) × 3° (length). The outer ends of the rectangles were rounded by adding a 2° × 2° square, which had its corners rounded by creating a distance co-ordinate (*z*) using the following equation:

$$z = \sqrt{{x}^{6}+{y}^{6}},$$

where *x* and *y* were vectors ranging from − 1 to 1 with a spacing of 0.025°. Pixels with *z* values of > 1 were removed to create the rounded corners. To create the four-rectangle BO-reverses mask we applied the same rounding to the inside edges of the mask. The duration of the target was always 67 ms, and the mask duration was always 133 ms.

#### Procedure

Each participant first performed a training session in which they viewed the masks and reported their perception of the two mask types. All participants freely reported that they perceived the BO-stable mask to consist of two overlapping transparent bars that formed a cross-shape and the BO-reverses mask to be four separate rectangles. After this they performed a brief training session of 28 trials in which they performed the contrast judgement task without any mask. They then performed the same training session in the presence of the BO-reverses mask at SOAs of 0 and 167 ms (56 trials). After completing the training sessions, they proceeded to four sessions of 392 trials each of the full experiment. They were encouraged to take breaks both within and between sessions. They completed a second experimental session, with a further four sessions on a separate day (typically within a week of the first session), to yield a total of 3,136 trials. Each experimental session lasted approximately 1 h.

#### Data analysis

The fraction of trials that participants chose the variable-contrast checkerboard at each SOA (ψ) were fit with cumulative normal psychometric functions using the Palamedes toolbox:

$$\psi \left(x,\alpha ,\beta ,\gamma ,\lambda \right)=\gamma +\frac{(1-\gamma -\lambda )\beta }{\sqrt{2\pi }}{\int}_{-\infty }^{x}exp\left(-\frac{{\beta }^{2}{(x-\alpha )}^{2}}{2}\right),$$

where *x* is the contrast of the checkerboard, α = threshold, β = slope, γ = guess rate, λ = lapse rate. All four parameters of the function (guess rate, lapse rate, threshold, and slope) were allowed to vary within predetermined limits. Guess and lapse rates could vary between 0 and 0.1. The slope was allowed to vary between 0.1 and 0.35 and threshold could vary between 25 and 50%. After fitting, we estimated the PSE as the point at which the fitted function ψ reached 0.5 using the inverse of the logistic function. The PSEs were used for between-participant comparisons. Outlying PSEs were removed by *z*-scoring the PSEs at each value of SOA and removing PSEs with absolute *z* values of greater than 2. This resulted in the removal of seven data points (5.8%). A linear mixed-effects model was used to analyse PSEs across participants with one fixed effect (SOA) and one random intercept term (Participant). SOA was treated as a categorical variable in this analysis, and the model was fit using restricted maximum likelihood estimation (REML) using ‘fitlme.m’ in MATLAB 2024b.

### Results

**Fig. 4:**
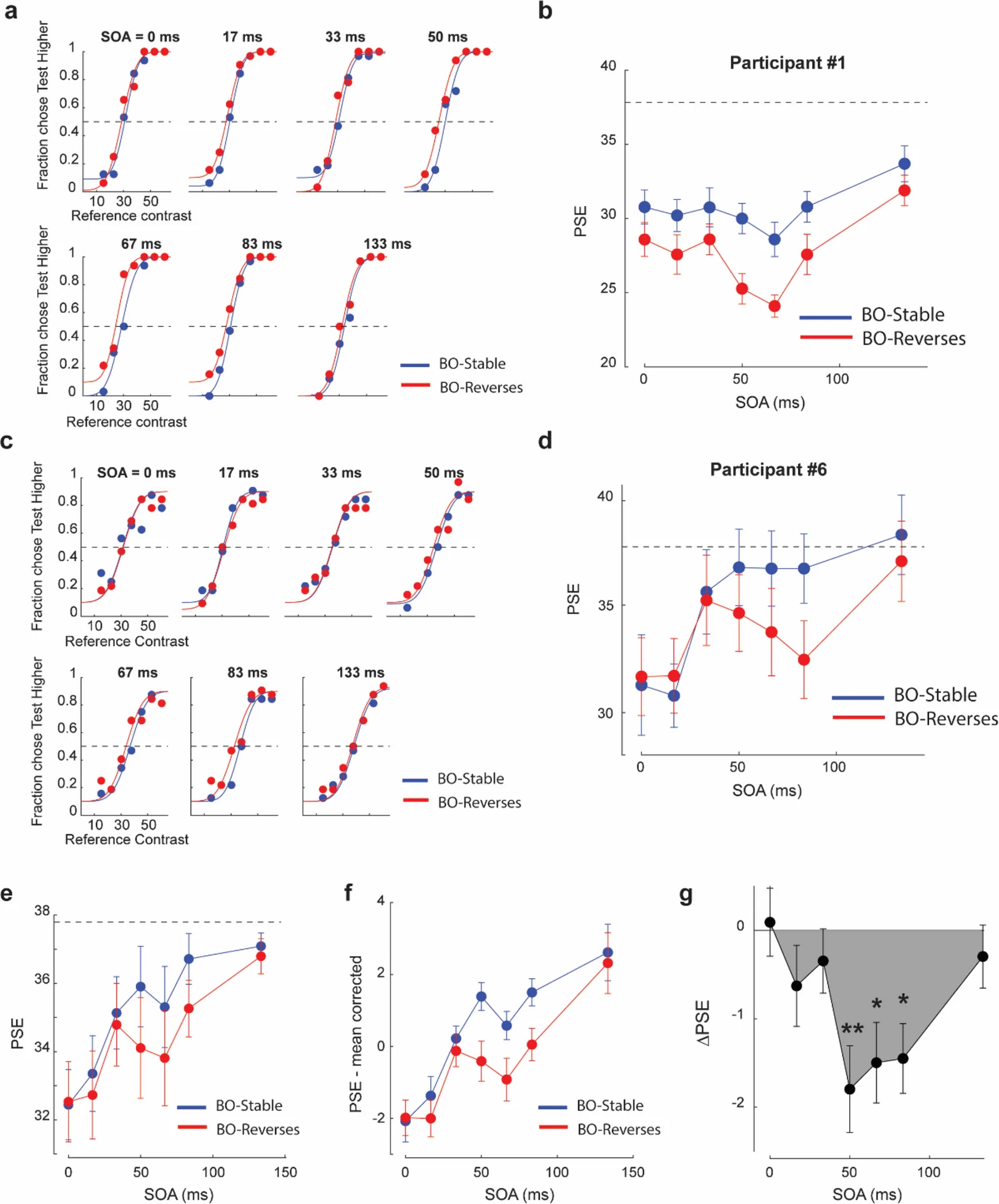
Experiment 2: BO reversal leads to stronger masking while using near-identical masks. **a** Example psychometric functions fit to the data of participant #1. **b** The PSEs from participant #1 showed a general negative offset compared with the physical contrast of the checkerboard (dashed line). A U-shaped decrease in perceived contrast was visible at the 50–83 ms SOAs. **c** Example psychometric functions fit to participant #6’s data. **d** PSEs from participant #6 showing an increasing trend with SOA. **e** Averages of the raw PSE across 16 participants. Error-bars indicate ± 1 *SEM*. **f** Average data after mean correction of each participant’s PSEs. The error-bars indicate ± 1 *SEM*. **g** The difference in PSE between the BO-stable and BO-reverses condition. The asterisks indicate the significance of a post hoc Wald test as carried out at each SOA (**p < 0.01, *p < 0.05, Bonferroni correction for multiple comparisons applied). (Colour figure online)

Psychometric functions were fit to each individual participant’s data at the seven tested SOAs and two mask types to estimate the physical contrast at which the variable-contrast checkerboard appeared similar in contrast to the constant-contrast checkerboard, which was subject to the effects of masking. The derived PSE was then compared between the two mask types. PSEs were generally lower than the physical contrast of the test checkerboard, indicating that the mask reduced the perceived contrast of the checkerboard. Participants varied in the effect of the masks on PSE. Some participants showed a typical Type B masking effect with a U-shaped reduction in perceived contrast at intermediate SOAs (e.g., Participant 1, Fig. 4a–b). The majority of participants showed a more complex relationship between SOA and PSE which resembled a combination of Type A and Type B effects (e.g., Participant 6, Fig. 4c–d). In these participants PSEs were lowest at the shortest SOAs regardless of mask type and gradually increased with increasing SOA. We ascribe this reduction in PSE at the shortest SOAS as being due to surround suppression of perceived contrast of the target checkerboard (see Discussion). On top of this increase in PSE with increasing SOA, we also observed a MM effect, visible as a U-shaped decrease in PSE that was present between SOAs of approximately 50–100 ms. Critically, the difference in PSE between the two mask types was unaffected by the surround suppression effect, as the two masks had very similar physical characteristics. As can be seen in the data of Participant 6, the BO-reverses mask caused a stronger reduction in PSE compared with the BO-stable mask in the critical SOA range for MM, but not at the shortest SOAs. This effect was also present across the population of 16 participants (Fig. 4e–g). Due to the underlying differences in the vertical offset of the masking curve between participants, the raw average PSEs were quite variable (Fig. 4e). We corrected for this difference by subtracting the average PSE across all conditions for each participant to create a mean-corrected PSE (Fig. 4f).

For statistical analysis we calculated the difference in PSE between the two mask types (ΔPSE) and examined how this varied with SOA (Fig. 4g). There was a significant effect of SOA on ΔPSE (LME model:* F*_6,99_ = 4.1, *p* = 0.001) which was driven by the lower PSEs in the BO-reverses condition in the 50–100-ms range. ΔPSE was significantly lower than zero for the 50 ms, 67 ms, and 83ms SOAs (*p* = 0.002, 0.01, 0.01 respectively, Bonferroni-corrected post hoc Wald test). The intercept of the model was not significant (*F*_1,99_ = 0.3, *p* = 0.58) suggesting that there was no overall offset in the effect of the two masks on the perceived contrast of the checkerboard.

### Discussion

The results indicate that the BO-reverses mask, which was perceived as four separate rectangles, produces a significantly stronger reduction in perceived contrast in the critical metacontrast SOA range than the BO-stable mask, which is perceived to be two transparent, overlapping rectangles. The physical difference between the two masks was small compared with the overall size of the mask and the difference in masking strength is unlikely to be related to these minor pixel-level differences (Fig. 3a). A more likely interpretation is that the small physical differences in the mask lead to a large difference in the perceived organisation of the mask—switching it from four discrete rectangles to two overlapping and transparent bars and we verified that each participant experienced this illusion prior to the start of the experiment. This difference in perceptual organisation results in a difference in the BO-assignment direction at the boundary between the checkerboard and the mask and this leads to the difference in masking strength. The results therefore support our theory that BO-reversals form a key component of the metacontrast masking effect and that stable and prolonged assignment of borders contributes to the perception of visual objects.

We note a few caveats here. Firstly, superimposed on top of the Type B metacontrast masking effect that differed between the masks was a Type A style masking effect that did not differ between the masks. The Type A effect led to reduced perceived contrast values at short SOAs (0–35 ms). A likely explanation for this effect are suppressive interactions between the contrast of the mask and the contrast of the target. Adding contrast to the surround of a target stimulus reduces the perceived contrast of the target (Chubb et al., 1989) and these interactions will be strongest during time periods when the two stimuli are concurrently present. This effect did not differ between the two mask types, as evidenced by a lack of difference between the mask types at short SOAs, suggesting that the masks were well controlled for physical contrast. We note, however, that the two masks were not identical—there were small differences due to the rounding of the inside corners of the BO-reverses mask. These pixel-level differences were small compared with the overall size of the mask and the rounding of the corners reduced the physical contrast of the BO-reverses mask, which, if anything, should have reduced its effectiveness as a mask. In contrast, we found the BO-reverses mask to have the strongest metacontrast effect. Lastly, while this experiment demonstrates a clear reduction in perceived contrast when using the BO-reverses mask, the size of the masking effect was quite low. This was due to the nature of the mask, which was relatively low in contrast compared to the relatively high-contrast checkerboard targets. This arrangement was necessary to produce consistent perception of the BO-stable mask as transparent, but it resulted in masks that reduced perceived contrast by 2–3% rather than causing invisibility of the checkerboard target. Nevertheless, the study demonstrates the principle that metacontrast masks that reduce the visibility of a target can be rendered less effective by subtle manipulations of perceptual organisation that maintain stable BO relationships across the target–mask transition.

## General discussion

**Fig. 5:**
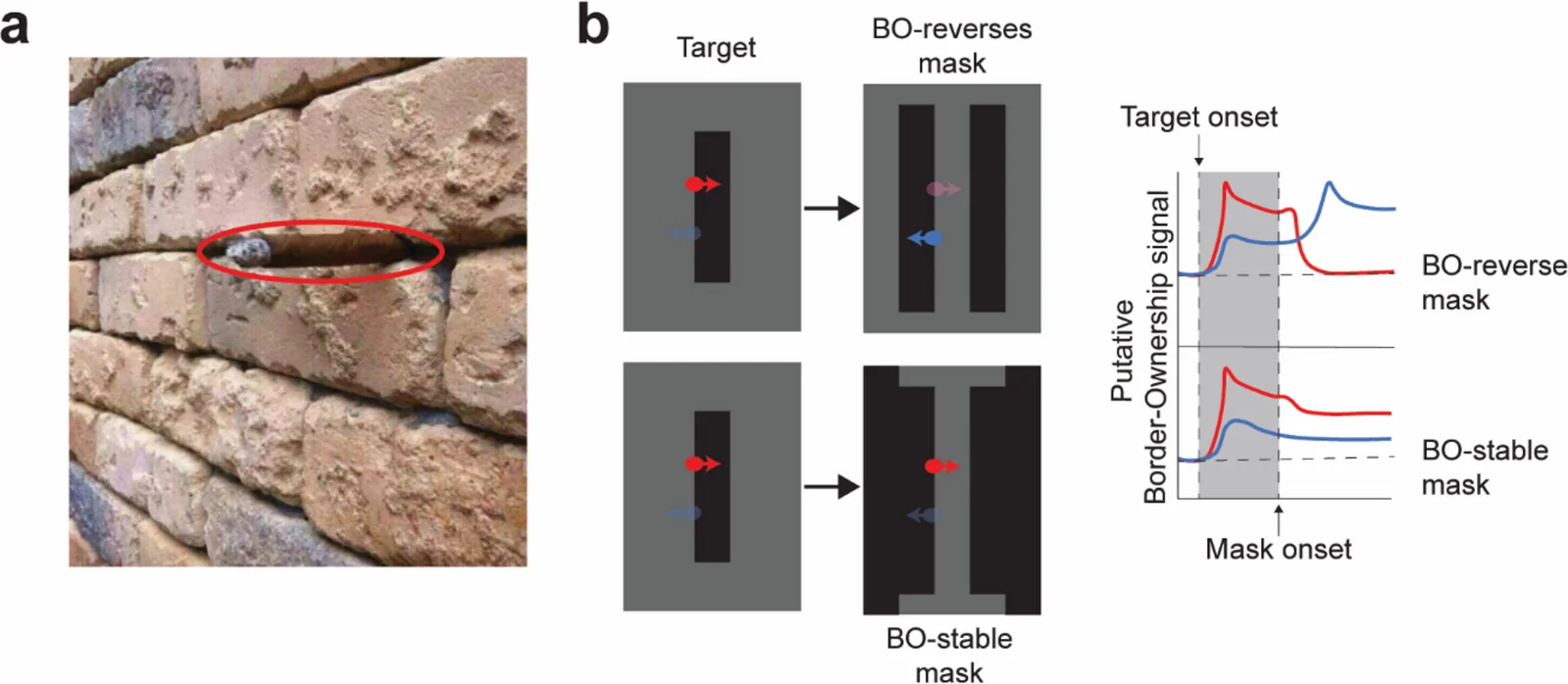
Theoretical BO neural activity during MM paradigms. **a** Solution to the illusion shown in Fig. 1a. A cigar is present in the wall. **b** The putative activity of rightwards preferring (red) and leftwards preferring (blue) BO cells on one border of the target stimulus in Experiment 1a. Rightwards preferring cells will be more active during the target period. Upon appearance of the BO-reverses mask, the activity of the two populations is rapidly reversed, potentially disrupting recurrent interactions with cells in early visual areas. The BO-stable mask preserves the direction of BO, and so the population activity will remain relatively constant across the target–mask transition, stabilising perception of the target. (Colour figure online)

Border-ownership is a critical process in the establishment of a 3D perceptual model of the world, and image regions that fail to gain ownership of their borders can remain unrecognised. In the ‘cigar-illusion’, the cigar is initially invisible to most observers as its borders are assigned to the surrounding bricks. Typically, the cigar remains invisible until top-down instructions are given about the location and identity of the object (Fig. 5a). This extreme example shows that failure to gain BO can render an object permanently invisible. We have demonstrated that BO assignment plays a critical role in the perception of briefly presented objects during MM. Across both experiments, masks that reversed ownership of the target’s borders decreased the visibility of target shapes compared with masks that maintained the direction of ownership. In Experiment 1, BO-reversing masks reduced the ability of participants to make length judgements about the targets whereas in Experiment 2 they reduced contrast perception. In both experiments masks that preserved the direction of BO had much less effect on these perceptual measures. We therefore propose that a key underlying mechanism of MM is the reversal of BO at the target–mask boundary. The reversal of BO is present in many metacontrast masking paradigms (Breitmeyer & ÖĞmen, 2006). Typical metacontrast masks, such as an annulus that surrounds a circular target or two bars that flank a central target bar (Kahneman, 1967; Macknik & Livingstone, 1998) are configured so that the inner boundary of the mask reverses the direction of border ownership at the target–mask interface. This suggests that at least part of the effectiveness of the masks in these paradigms is due to the mask reversing, or ‘stealing’, ownership of the target’s borders.

Metacontrast paradigms that investigate the perceptual properties of the target have a U-shaped relationship with SOA in which the strongest masking effects are observed at intermediate SOAs in the 30–100-ms range (Kahneman, 1967; Kolers, 1962; Weisstein & Haber, 1965). At short SOAs, the target and mask are present simultaneously on the screen and can form grouped or ambiguous percepts. During this phase the BO relationship between the target and mask is ambiguous and the mask is ineffective. In our studies the difference between the two mask types was strongest if the mask appeared at the same time as the target disappeared, at an SOA of 50 ms. At this SOA, the target and mask did not temporally overlap and the appearance of the BO-reverses mask immediately reversed the BO relationships at the target–mask boundary. We did not investigate different combinations of target duration and SOA and it remains to be determined whether the concurrent disappearance of the target and appearance of the mask is necessary for the strong difference between the mask types in our paradigm. However, we also observed clear masking effects, and differences between the mask types, at longer SOAs which have an interstimulus interval between the target and mask. This suggests that the encoding of BO direction may be robust to brief intervals during which the target is not present (O’Herron & von der Heydt, 2009).

In our experiments the participants made discriminative judgements about stimulus attributes (i.e., length and contrast) rather than having to detect the target. Metacontrast masks typically affect perception of the surface properties of objects, such as colour or contrast, and it has long been recognised that the mask has differential effects on the perception of the borders and surface properties of the target (Breitmeyer & Tapia, 2011; Kolers, 1962; Paradiso & Nakayama, 1991). The experimental design did not allow us to discriminate between perceptual effects on surface properties and effects on the ability to detect the target. Our paradigm required carefully matched masks and pilot work suggested it was difficult to find a mask–target design that allowed control of BO while producing Type B effects on target-detection ability. Future studies can investigate whether this is because the BO-reversal primarily affects perception of the surface properties of the object while leaving contour detection, and therefore target detection, intact (Breitmeyer & Tapia, 2011; Breitmeyer et al., 2006; Lamme et al., 1999; Scholte et al., 2008; Self et al., 2013).

How does the BO-reversal based theory of MM relate to previous models? In both Experiments 1 and 2, the luminance contours at the target–mask interface were very closely matched in physical appearance for the two mask types. In Experiment 1, the luminance change induced by the mask was identical at the target–mask boundary but differed further away from the target, whereas in Experiment 2 we used a near identical mask in both conditions, matching the physical contours of the two mask types as closely as possible. Despite this, masking was stronger when the mask gained ownership of the target’s borders. For this reason, any theory of MM which requires inhibitory interactions between the contours of the target and the mask cannot explain our findings, as the contours were similar for both mask types. Our results would not be predicted by the dual-channel inhibition theory of MM, which proposes that the transient activity initiated by the onset of the backward mask interrupts the sustained activity of an earlier presented target, thereby decreasing its visibility (Breitmeyer & Ganz, 1976; Breitmeyer et al., 2004; Ogmen et al., 2003). In our experiments the transients produced by the masks were carefully matched between conditions and cannot therefore explain the difference in visibility of the targets. Our results instead support ideas that the effect of masking on perception of the target is due to its effects on later reentrant processing stages and figure–ground assignment processes (Fahrenfort et al., 2007; Lamme et al., 2002; Ro et al., 2003). Specifically, we propose that the BO-reverses mask causes a rapid curtailment of BO-tuned neural activity (Fig. 5b) supporting the perception of the target region as figural through recurrent interactions with early visual areas, leading to impaired perception of the target. In contrast the BO-stable mask maintains BO-tuned activity allowing prolonged recurrent interactions with early visual areas, promoting visibility of the target. Our theory therefore also shares some conceptual similarities with theories proposing that masks are particularly efficient if there is a mismatch between reentrant processing of the target shape and the new, feedforward information provided by the mask (e.g., *object substitution* theory; Di Lollo et al., 2000; Enns & Di Lollo, 1997). In the BO-reverses conditions, there is a mismatch between the reentrant information indicating that the target region is a figure, and the incoming activity generated by the mask, which indicates that the target region is a background. In our experiments the decrease in target visibility could be due to a mismatch between feedback from BO-tuned cells and incoming feedforward processes related to the mask borders. There is also some conceptual similarity with computational models of MM in which sustained border processing is necessary for target visibility (Francis, 1997); however, our proposal utilises the known properties of BO tuned neurons in cortex which were not discovered until after the development of the above model.

At the neuronal level, Zhou et al. (2000) showed that the perceptual assignment of borders to figures is reflected in the activity of cells in early visual cortical areas of monkeys, particularly in V2. *BO-cells* are neurons that encode the side to which a border belongs (von der Heydt & Zhang, 2018; Zhou et al., 2000) and can be found in large numbers in V2, V3, and V4 (Franken & Reynolds, 2021; Hesse & Tsao, 2016; Jeurissen et al., 2024; Zhou et al., 2000). These cells engage in recurrent interactions with cells in V1 leading to the establishment of segmented visual objects in perception (Jeurissen et al., 2024; Kirchberger et al., 2021). We propose that sustained and consistent activation of a population of BO-tuned cells is necessary for establishing a percept through engagement of recurrent circuits. In conditions in which the direction of BO is consistent across the target–mask transition, there will be a more sustained activation of a population of BO cells. For example, in Fig. 5b the BO-cells that prefer the border that is owned by the target bar (in red) will have a stronger response than those that encode that the border that would be owned by the surrounding background region (in blue). After the appearance of the ‘BO-reverses’ mask the neural activity of the two populations will reverse, whereas in the ‘BO-stable’ condition the relative activity of the two populations will remain the same. Previous studies have shown that the presentation of new objects that reverse BO status result in rapid reversals in neural activity patterns in mid-tier visual areas, whereas transitions to ambiguous borders result in a slower decay of activity (O’Herron & von der Heydt, 2009; O’Herron & von der Heydt, 2011). In our study the interstimulus interval did not contain an ambiguous border, so it is unclear how BO-cells would respond during the ISI. It is likely that, in the absence of feedforward border information, BO cell activity would begin to decay towards spontaneous activity but during this decay the residual BO activity may be enough to sustain recurrent interactions. Maintained activity levels of BO-cells ‘pointing’ to the target region in the BO-stable condition could allow more time for the establishment of recurrent interactions between neurons at different levels of the visual hierarchy which is thought to be critical for the establishment of a visual percept (Fahrenfort et al., 2007, 2017; Kirchberger et al., 2021; Lamme et al., 2002; Xin et al., 2025). In contrast, in the BO-reverses condition the sudden curtailment of activity of BO cells supporting the figural status of the target will result in shorter periods of recurrent activity between hierarchical levels and less time for the target to be established as a percept. We note that many BO-tuned cells are also sensitive to the contrast polarity of the border (Zhou et al., 2000), and in our stimuli the contrast polarity reversed across the target–mask transition. Future studies could investigate whether masks that maintain both the BO-relationships and the contrast polarity may be even weaker than those studied here as the same population of BO-tuned cells will remain active across the target–mask transition.

In summary, our results demonstrate that the accuracy and strength of a visual percept depend on a sustained and consistent representation of border-ownership. Reversals in BO contribute to the efficacy of metacontrast masks, highlighting the stability of the brain’s internal model of the world as an important factor in conscious perception.

## Data Availability

The raw data accompanying this manuscript can be downloaded from Zenodo (https://doi.org/10.5281/zenodo.22251944).

## Declarations

### Open practices statement

The data and code for our study are shared by the lead contact upon request, and none of the experiments were preregistered.
